# Addendum: CYTOPLASMIC FILAMENTS OF *AMOEBA PROTEUS*: I. The Role of Filaments in Consistency Changes and Movement

**DOI:** 10.1083/jcb.46.2.26701082026a

**Published:** 2026-01-22

**Authors:** Thomas D. Pollard, Susumu Ito

Vol. 46, No. 2 | https://doi.org/10.1083/jcb.46.2.267 | August 1, 1970

Our 1970 *JCB* paper (https://doi.org/10.1083/jcb.46.2.267) documents a direct connection between actin filaments and cytoplasmic movements. The primary data showing movements in cell-free extracts of *Amoeba proteus* were recorded on 16-mm movie film and shown at the 1968 Annual Meeting of the American Society for Cell Biology (ASCB; Pollard and Ito, 1968). However, no technology was available at the time to include this data in the paper. This addendum includes eight video sequences from those movies.

## Background modified from the paper

Although cytoplasmic streaming is a basic property of cells, the mechanism of the movement of subcellular particles is not clearly understood. Since cytoplasm isolated from plant cells and amoebas exhibited independent motility (Jarosch, 1956; Allen et al., 1960; Thompson and Wolpert, 1963), the motive force must be generated in the cytoplasm. The structures thought most likely to produce movement are the two types of cytoplasmic filaments: (a) solid filaments, which occur in several sizes, and (b) hollow microtubules. Thin filaments, about 75 Å in diameter, and thicker filaments, 150 Å in diameter and up to 0.5 µm long, were discovered in electron micrographs of thin sections of giant amoebas whose plasma membranes were torn before fixation (Nachmias, 1964) or extracted with glycerol (Schäfer-Danneel, 1967). Glycerinated amoebas contracted slightly upon adding ATP (Schäfer-Danneel, 1967).

**Figure:**
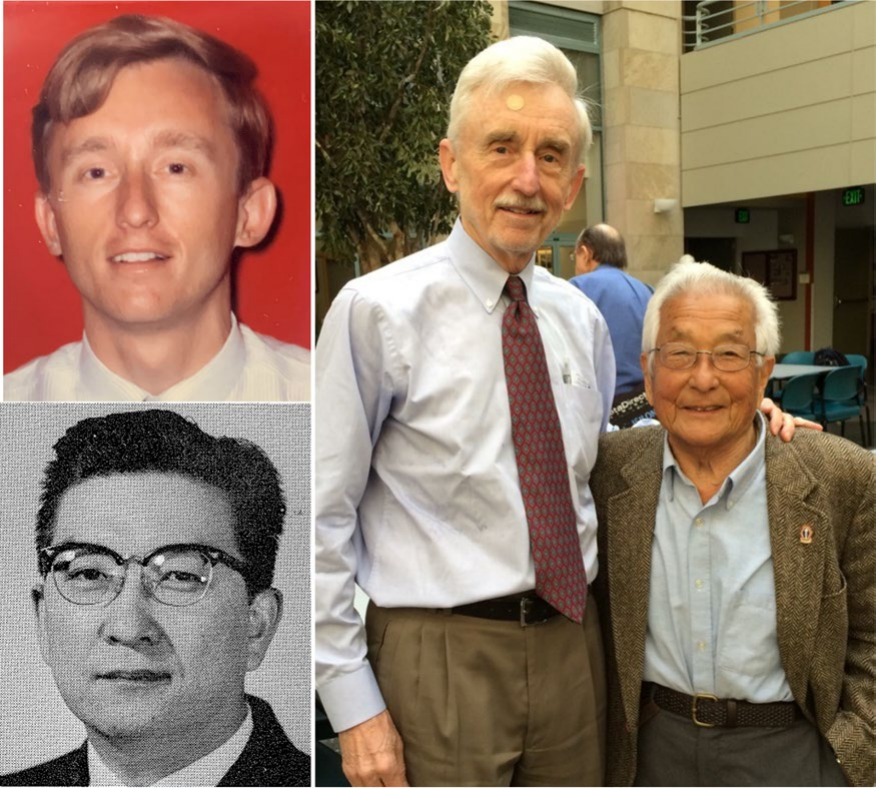
The authors in 1968 (left) and in 2015 a few months before Sus Ito passed at age 95 (right).

We did our work in the spring of 1968 and showed our movies and electron micrographs at the 1968 ASCB meeting. Unknown to us at that time, Sadshi Hatano and Fumio Oosawa (1966) had isolated actin from *Physarum polycephalum*. Subsequently, Weihing and Korn (1969) isolated actin from *Acanthamoeba*, and Hatano, Kondo, and Miki-Noumura (1969) isolated actin from sea urchin eggs.

We worked on the carnivorous giant amoeba called *Amoeba proteus*, which moves rapidly by vigorous cytoplasmic streaming (Video 1). Inspired by the success of Hugh Huxley using electron microscopy to understand the contractile apparatus of striated muscle (Huxley, 1957), we initially tried a purely anatomical approach, which failed. Fortunately, we learned about work by Thompson and Wolpert (1963), who reported that a crude cytoplasmic extract from *Amoeba proteus* exhibited vigorous movements when ATP or ADP was added and it was warmed from 4° to 20°C. We did not know that others had tried but failed to confirm these findings.

## Methods modified from the paper

We grew large-scale cultures of *Amoeba proteus* in a dilute buffer with living *Tetrahymena pyriformis* as food. We modified the method of Thompson and Wolpert (1963) to prepare the motile fraction of cytoplasm. We cooled amoebas to 4°C for 12–24 h and concentrated them by low-speed centrifugation. 5 cc of cell slurry were centrifuged at 4°C for 10 min at 18,000 rpm in a Beckman Type SW 39L rotor. The centrifugal force of 35,000 *g* at the tip of the tube fragmented the cells into four distinct layers. The second layer (about 1–2 ml) consisted of membrane-bounded bags of cytoplasm. We mixed this layer with an equal volume of glass-distilled water or Tris-maleate buffer, pH 7.0 (10 or 20 mM), and lysed the cells with five gentle strokes of a Teflon-glass homogenizer. We removed large fragments of plasma membrane by centrifugation at 1,000 *g* for 5 min. The supernatant was called Extract 1.

## Observations by phase-contrast microscopy

The original paper did not include the conditions used to make the movies, as no way existed in 1968 to share the movies with the publication. We used a Zeiss phase-contrast microscope to make movies on 16-mm film at 30 frames per second. In 2015, I digitized a sample of these movies. The movies are played back in these videos at the same rate. Thus, all sequences are in real time. Our *JCB* paper did not list the optics we used, but I am confident that most of the movies were made with a 100x objective. Magnifications are approximate, based on the sizes of membrane-bound particles in the movies and electron micrographs in our paper.

**Figure:**
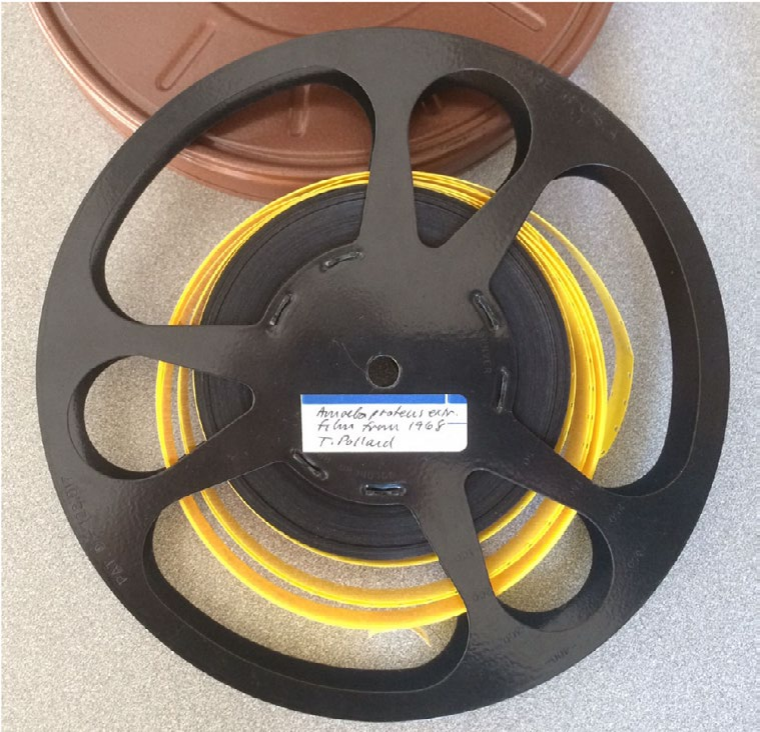
Reel of 16-mm film with movies of movements in extracts of *Amoeba proteus*.

**Video 1.** *Amoeba proteus* moving in real time on a glass microscope slide (23 s, sequence 200).

**Video 2.** **Preparation of motile cell extracts.** Scene 1: Homogenate of cold cells with large sheets of plasma membrane suspended in cytoplasm (10 s, sequence 1700). Scene 2: Supernatant after centrifuging cold homogenate at 1,000 *g* for 5 minutes to remove large membrane structures. The supernatant contained small organelles including mitochondria. These cytoplasmic “particles” underwent Brownian movements but not directed movements (20 s, sequence 2300).

**Video 3.** **Movements of particles in Extract 1 sealed under a coverslip with 2 mM ATP and warmed to 22°C.** Scene 1: Within 30–180 seconds, individual particles started to make saltatory (short, linear) movements, followed by larger groups of particles moving in unison while held in a semirigid structure invisible in the phase-contrast and polarizing microscopes (10 s, sequence 4892). Scene 2: This area of the extract made vigorous ripping and pulling motions of particles, similar to cytoplasmic streaming (15 s, sequence 5347). Scene 3: This region of the extract had violent movements as regions contracted and retracted from each other, concentrating particles in apparently gelled areas at the end (18 s, sequence 4250).

**Video 4.** **Late-stage movements of Extract 1 sealed under a coverslip with 2 mM ATP and warmed to 22°C.** Scene 1: Part of a large ring of gelled extract constricted toward its center located out of the field of view to the right. Note particles moving on fibrils trailing behind the constricting ring (23 s, sequence 8020). Scene 2: Lower magnification to show regions of the extract condensed into gelled strands moving on the glass surface (15 s, sequence 8020).

## Observations by electron microscopy of thin sections of fixed and embedded extracts

Modified from the abstract to the paper: At 0°C, this extract was nonmotile and similar in structure to amoeba cytoplasm, consisting of groundplasm, vesicles, mitochondria, and a few short filaments 160 Å in diameter. The extract underwent striking ATP-stimulated streaming when warmed to 22°C. We observed two phases of movement. During the first phase, the apparent viscosity usually increased, and numerous 50–70 Å filaments appeared in samples of the extract prepared for electron microscopy, suggesting that the increase in viscosity was caused, at least in part, by the formation of these thin filaments. During this initial phase of ATP-stimulated movement, these thin filaments were not detected by phase-contrast or polarization microscopy, but later, in the second phase of movement, 70 Å filaments aggregated to form birefringent microscopic fibrils. Centrifugation of Extract 1 at 10,000 *g* for 10 minutes produced Extract 2 consisting of pure groundplasm with no 160 Å filaments or membranous organelles. Extract 2 exhibited little or no ATP-stimulated movement, but 50–70 Å filaments formed and aggregated into birefringent fibrils. This observation and the structural relationship of the 70 Å and the 160 Å filaments in the motile extract suggested that both types of filaments may be required for movement. These two types of filaments, 50–70 Å and 160 Å, are also present in the cytoplasm of intact amoebas. Fixed cells could not be used to study the distribution of these filaments during natural amoeboid movement because of difficulties in preserving the normal structure of the amoeba during preparation for electron microscopy.

A subsequent paper identified the 70 Å filaments as actin filaments by decoration with myosin heads (Pollard and Korn, 1971).
